# Editorial: Community series in advances in pathogenesis and therapies of gout, volume II

**DOI:** 10.3389/fimmu.2025.1556844

**Published:** 2025-02-04

**Authors:** Lihua Duan, Jixin Zhong, Ye Yang, Yuan Liu, Xiaoxia Zhu

**Affiliations:** ^1^ Department of Rheumatology and Clinical Immunology, Jiangxi Provincial People’s Hospital, The First Affiliated Hospital of Nanchang Medical College, Nanchang, China; ^2^ Department of Rheumatology and Immunology, Tongji Hospital, Tongji Medical College, Huazhong University of Science and Technology, Wuhan, China; ^3^ Department of Medicine, University of Florida, Gainesville, FL, United States; ^4^ Department of Rheumatology and Clinical Immunology, the First Affiliated Hospital of Xiamen University, Xiamen, China; ^5^ Division of Rheumatology, Huashan Hospital, Fudan University, Shanghai, China

**Keywords:** gout, inflammasome, hyperuricemia, monosodium urate (MSU) crystal, cardiovascular disease

Gout is a common sterile inflammatory disease caused by abnormal purine metabolism (1). Uric acid is the final product of purine metabolism in the human body. The pathogenesis of gout involves the formation and deposition of monosodium urate (MSU) crystals in tissues due to elevated serum uric acid. MSU is recognized and phagocytic by macrophages, and subsequently activates the inflammasome NOD-like receptor thermal protein domain associated protein 3 (NLRP3), produces interleukin (IL)-1β and promotes the release of other pro-inflammatory factors and the aggregation of neutrophils, thereby triggering local or even systemic inflammatory responses (2). With the improvement of living standards and the increase in purine intake, the incidence of hyperuricemia and gout is increasing annually (3). It is worth noting that hyperuricemia has become an independent risk factor for various systemic diseases, especially cardiovascular diseases and chronic kidney diseases (4, 5). To further identify new strategies for the prevention and improvement of hyperuricemia as well as gout, this Research Topic exhibits a number of original research articles on the topic of advances in diagnosis, genetic involvement, pathogenesis, and comorbidities of hyperuricemia and gout.

In this Research Topic, He et al. explored the relationship between the Oxidative Balance Score (OBS, composed of scores for 20 dietary and lifestyle factors) and hyperuricemia/gout. Among adult participants in the National Health and Nutrition Examination Survey (NHANES) spanning from 2009 to 2018, higher OBS was found to be associated with a decreased risk of developing hyperuricemia/gout, underscoring its potential in the prevention and management of these conditions. Life’s Essential 8 (LE8) is a comprehensive measure of cardiovascular health promoted by the American Heart Association, and Wang et al. suggested that higher LE8 scores are robustly associated with lower odds of hyperuricemia.

Recent reports have suggested that the intestine may play a crucial role in the excretion of uric acid outside the kidneys (6). Yang et al. performed a large Taiwanese population study to examine the risk factors for self-reported peptic ulcer disease (PUD), and found that hyperuricemia was associated with low prevalence of self-reported PUD in males, but not in females. Gouty nephropathy (GN) is a renal condition caused by precipitation of MSU in the kidney tubules (7). Li et al. introduced a new approach for the induction of GN by intrarenal injection of MSU, which may potentially serve as an experimental groundwork for future studies on the pathogenesis and prevention strategies of GN. Uric acid excretion in the intestine and kidney is closely related to the polymorphism of ABCG2 gene. Many common variants associated with gout have been reported, e.g., rs22331142 in *ABCG2* in a Taiwanese population (8). Nevertheless, Tseng et al. identified the rare variants rs559954634, rs186763678, and 13-85340782-G-A for the first time to be associated with gout in Taiwanese male, and the mechanism of these rare variants is worthy of further study.

The above-mentioned excessive intake of purine and the excretion disorder of uric acid lead to the formation of MSU, which participate in pyroptosis, to activate NLRP3 inflammasome of innate immune cells. IL-38 is a newly discovered anti-inflammatory cytokine, and a cardiovascular study highlighted that IL-38 inhibits the activation of the NLRP3 inflammasome (9). Huang et al. reported in this Research Topic that the serum levels of IL-38 were reduced in patients with gout compared to that in negative controls, suggesting that IL-38 may have immunomodulatory effects on gout inflammation and possesses clinical application value. In addition, Absent in melanoma 2 (AIM2) inflammasome stimulates apoptosis-associated speck-like protein containing a CARD (ASC) to facilitate the oligomerization and subsequent proteolytic maturation of pro-caspase-1 (10). Chu et al. investigated the action of AIM2 in the inflammatory processes of acute gouty arthritis (AGA) and asymptomatic hyperuricemia(AHU), which provide new insights into the involvement of the AIM2 mediated pyroptosis pathway in the development of AGA and strategies for treating gout.

In addition to inflammasomes, there are more biomarkers involved in the pathogenesis of gout. Using the gout-associated transcriptome dataset GSE160170, Wang et al. found that there were significant differences in the expression levels of CXCL8, CXCL1, and CXCL2 between the gouty group and the healthy group, and they also predicted 10 chemicals related to these proteins. Utilizing a high-quality, high-throughput proteomic analysis technique, Huang et al. identified differentially expressed proteins in the serum and synovial fluid of gout patients in comparison to that of healthy controls and osteoarthritis. These discoveries are potential biomarkers for diagnostic purposes and are believed to have critical roles as pathogenic factors in the pathophysiology of gout.

Overall, the original research articles in this Research Topic cover a series of important aspects in the field of potential biomarkers, influencing factors and clinical management of gout, which may provide new insights into the diagnosis and intervention of gout and hyperuricemia.
